# A Positive Legacy of Trauma? The Role of Perceived Social Support on Mental Health Among Earthquake Survivors During the COVID-19 Outbreak

**DOI:** 10.3389/fpsyt.2022.845929

**Published:** 2022-04-27

**Authors:** Dongfang Wang, Shuyi Zhai, Jiaqiao Chen, Yao Chen, Shiming Hua, Chengchen Wang, Fang Fan, Xianchen Liu

**Affiliations:** ^1^Guangdong Key Laboratory of Mental Health and Cognitive Science, Ministry of Education Key Laboratory of Brain Cognition and Educational Science, Centre for Studies of Psychological Applications, School of Psychology, South China Normal University, Guangzhou, China; ^2^Faculty of Medicine, McGill University, Montreal, QC, Canada; ^3^Center for Public Health Initiatives, University of Pennsylvania, Philadelphia, PA, United States

**Keywords:** perceived social support, depression, anxiety, trauma exposure, COVID-19

## Abstract

**Background:**

People with prior experience of severe trauma may be particularly vulnerable in the face of the COVID-19 pandemic. However, little is known about mental health problems among prior trauma survivors during the pandemic outbreak.

**Methods:**

A total of 362 Wenchuan earthquake survivors were assessed using Patient Health Questionnaire, Generalized Anxiety Disorder Scale, as well as Multidimensional Scale of Perceived Social Support, as part of an online survey between February 3 and 10, 2020.

**Results:**

Our results showed that 6.6 and 4.7% of the participants experienced depression and anxiety during the COVID-19 outbreak, respectively. Perceived social support was negatively associated with depressive and anxiety symptoms. Earthquake exposure has no direct effect on current depressive and anxiety symptoms, but it would moderate the direct relationship between perceived social support and psychological symptoms.

**Conclusions:**

Our findings suggested that trauma exposure may lead to salutogenic outcomes. The protective effect of perceived social support on psychological symptoms was greater in people with a higher level of trauma exposure than in a lower one.

## Background

Numerous studies have shown that the COVID-19 pandemic leads to the development of mental health problems in public (1, 2). For some special groups, such as psychiatric patients (3), frontline healthcare workers (4), and even patients infected with COVID-19 (5), their mental health is even more likely to be affected by the pandemic. However, little attention has been paid to the mental health of another high-risk group during the pandemic: survivors of prior trauma.

Theoretically, prior trauma exposure and subsequent posttraumatic stress (PTS) symptoms may intensify one's vulnerability when facing additional stressors. By draining one's resources (6, 7) and coping capacity, trauma exposure and PTS symptoms heighten one's sensitivity to stress (8, 9). One late study in a sample of 976 adults indicated that history of trauma exposure and resultant PTS were associated with an elevated risk for psychological distress following COVID-19 (10). This finding leads us to suspect that whether individuals who experienced natural disaster (e.g., earthquake) could have greater risks of poor mental health during the COVID-19 pandemic.

Considered as an important disaster preparedness resource, social support is linked to better mental health outcomes for survivors after disasters (11). More specifically, it has been proposed that stronger social support can predict better mental health functioning (12, 13), enhance resilience to stress, and help protect against developing trauma-related psychopathology (14). Meanwhile, people who give and receive social support before the occurrence of disasters are significantly less likely to develop mental health problems (e.g., depressive symptoms) during the post-disaster time compared to those without support (15).

On the other hand, perceived social support is distinct from received social support, which is a better predictor of mental health and support utilization than other measures (16). People suffering from a greater degree of disaster-related traumatic stressors are more likely to seek and receive greater amount of actual support, which consists of a significant indirect path to reduce distress. Greater received support predicted greater perceived support over time, and greater perceived support in turn predicted greater reductions in distress over time, although perceived support tends to decrease over time (17–21). Furthermore, the level of perceived social support was negatively impacted by disaster-related stressors as well as subsequent life stressors in the aftermath of disasters (22). Accordingly, what about prior trauma survivors' perceived social support while facing a subsequent traumatic events such as the COVID-19 pandemic?

In the light of the foregoing discussion, we conducted the present study to examine the level of perceived social support and the prevalence of mental health problems among Wenchuan earthquake (2008) survivors during the COVID-19 outbreak. Three specific objectives were as follows: (a) to investigate depression and anxiety prevalence rates among 362 earthquake survivors during the pandemic outbreak; (b) to examine the effect of perceived social support in relation to depression and anxiety; and (c) to explore the differences in the relationship between perceived social support and mental health among survivors who suffer from different levels of earthquake exposure severity. It was hypothesized that perceived social support would be negatively associated with depression/anxiety. Earthquake exposure severity would moderate the direct association between perceived social support and mental health. Specifically, people suffering from greater degree of earthquake exposure are more likely to perceive greater social support, leading to fewer mental health problems.

## Methods

### Participants and Procedure

The 8.0-magnitude earthquake occurred on May 12, 2008, in Wenchuan county of Sichuan province, which has been the strongest earthquake over the past 50 years in China. The earthquake was devastating: 69,197 died, 374,176 were injured, and 18,222 were missing. Meanwhile, at least 4.8 million residents were left homeless due to their houses being destroyed by the earthquake. Fan et al. conducted a longitudinal study of mental health among adolescent survivors exposed to the Wenchuan earthquake in May 2008 (23). A total of 1,573 Wenchuan earthquake survivors completed assessments of mental health at 6 months after the earthquake (sampling time: November 2008) (23). Among these participants, 410 completed the web-based survey during the COVID-19 outbreak (sampling time: from February 3 to 10, 2020), and the response rate was 26.1%. To control the quality of the survey responses, exclusion criteria included was that “missing information >25%” and “response time <5 min.” Finally, 362 participants were included in the subsequent analyses. The chi-square test and *t*-test were used to compare the participants who participated in the web-based survey during the COVID-19 outbreak with those whose did not in major variables at baseline. There was no significant difference in age (*t* = −1.05, *p* = 0.292) and earthquake exposure (*t* = −0.79, *p* = 0.431) between these two groups. Men were less likely to participate (χ^2^ = 15.90, *p* < 0.001), mainly because they were more likely to drop out at school age. Among participants, 93.6% (*N* = 339) of the survivors lived in Sichuan Province during the survey period, which was a low infection risk area with <1,000 cumulative confirmed cases during the COVID-19 outbreak (24).

Researchers sent the informed consent and a specific web link or quick response (QR) code to participants through their contact information (e.g., QQ, WeChat, or SMS). Participants completed the online survey by clicking the questionnaire link or scanning the QR code of the questionnaire with mobile phones. This study was entirely voluntary; interested participants needed to sign an electronic informed consent form before the survey and could quit at any time. The ethics board of the South China Normal University (SCNU-PSY-2020-01-001) examined and approved the project. Participants were also provided psychological counseling from the School of Psychology, South China Normal University. If needed, participants can also assess free online psychological counseling service (“Xin-Qing”Hotline) from the School of Psychology of South China Normal University.

### Measures

#### Sample Characteristics and Trauma Exposure

Sample characteristics included sex, age, marital status, family income, history of mental and physical illness, history of smoking, and alcohol use.

The main two trauma exposures in this study are Wenchuan earthquake and COVID-19 pandemic-related factors. Earthquake exposure was assessed using four items (25): I1: death, injury, and/or missing of family members; I2: house damage; I3: property loss; I4: witness or hearing of tragic scenes. Each item was rated on a five-point Likert scale with 1 representing the lowest level of exposure and five representing the highest. Summing up scores on all items generates a total score, indicating overall severity of earthquake exposure.

Pandemic-related factors were assessed using three questions: Q1: Are there confirmed or suspected cases in your community or village? (1 = yes, 0 = no); Q2: Do you have relatives or friends who have been infected with COVID-19? (1 = yes, 0 = no); and Q3: How much time are you exposed to news and information about COVID-19 on social media? (1 = <1 h/day, 2 = 1–2 h/day, 3 = >3 h/day).

#### Perceived Social Support

The Multidimensional Scale of Perceived Social Support (MSPSS) was used to assess participants' perceived social support (26). It consisted of 12 items addressing the following three domains: family, friends, and significant others. Each item was scored on a 7-point scale ranging from 1 (very strongly disagree) to 7 (very strongly agree), with a range of 12–84. A higher total score indicated greater level of perceived social support. Degree of social support can be determined by the following cuto? scores: 12–48 low social support, 49–68 moderate social support, and 69–84 high social support. The Chinese version of MSPSS was reported to have good reliability and validity (27). It also had satisfactory internal consistency in this study (Cronbach' α = 0.95).

#### Depressive Symptoms

The 9-item Patient Health Questionnaire (PHQ-9) was used to assess participants' depressive symptoms over the past 2 weeks (28). Each item was answered on a 3-point Likert scale (0 = not at all, 1 = several days, 2 = more than half the days, and 3 = nearly every day), with higher scores indicating higher levels of depressive symptoms. The cutoff point of 10 was usually used for demonstrating clinically significant depression (29). The Chinese version of PHQ-9 has been reported to have good reliability and validity in the Chinese sample (30). In the current sample, Cronbach's α for PHQ-9 was 0.89.

#### Anxiety Symptoms

The 7-item Generalized Anxiety Disorder (GAD-7) was used to measure participants' anxiety symptoms over the past 2 weeks (31). Responders should provide a response for each item using a 6-point scale ranging from 0 (not at all) to 3 (nearly every day). Summing up scores on all items would generate a total score indicating the overall severity of anxiety symptoms. A preliminary study suggested that a cutoff score of 10 is the optimal threshold to indicate clinical level of anxiety (32). The scale of the Chinese version has demonstrated satisfactory psychometric properties in the Chinese population (33). In the present study, GAD-7 also demonstrated high internal consistency, with the Cronbach's α being 0.93.

#### Data Analyses

All statistical analyses were conducted using SPSS, version 23.0, and *p* < 0.05 were considered statistically significant for all two-tailed tests. Descriptive statistics were calculated for sample characteristics, pandemic-related factors, and earthquake exposure. To assess the differences between levels of perceived social support in relation to PHQ-9 and GAD-7, χ^2^-test and one-way ANOVA were used, as appropriate. Pearson correlations were examined among earthquake exposure, MSPSS, PHQ-9, and GAD-7. Meanwhile, PROCESS was used to examine the mediation hypotheses, with 5,000 iterations to estimate the effect size of models (34). Harman's one-factor test was conducted to examine common method variance before regression analysis (35). The moderation effect was tested: MSPSS score was entered as the predictor, earthquake exposure was entered as the moderator, and PHQ-9 or GAD-7 score was entered as the outcome. Simple slopes were calculated for high, medium, and low levels of earthquake exposure (using the mean score and cutoffs either one standard deviation above or below the mean), to determine the level at which perceived social support starts to have a significant correlation with earthquake exposure. Sample characteristics and pandemic-related factors were also included in the current analyses as covariates.

## Results

This sample consisted of 362 Wenchuan earthquake survivors, 125 men and 237 women. Their age ranged from 25 to 28 years old, with the average age of 26.41 (SD = 0.65) years; 1.4% participants lived in the community or village with confirmed or suspected cases, and 1.9% reported that their relatives or friends have been infected with COVID-19. Other sample characteristics and trauma exposure are listed in Table 1.

**Table 1:** Sample characteristics and trauma exposure.

**Variables**	**Characteristics**	***N***	**%**
**Socio-Demographic**			
Sex	Female	125	34.5
	Male	237	65.5
Age [years, M (SD)]	26.41 (0.65)		
Marital status (married)	Married	148	40.9
Family income (monthly)	<5,000 RMB	179	49.4
	5,000–10,000 RMB	122	33.7
	>10,000 RMB	61	16.9
History of mental disorders	Yes	3	0.8
Chronic physical illness	Yes	3	0.8
Smoking	Yes	71	19.6
Alcohol intake	Yes	134	37.0
**Pandemic-Related factors**			
Confirmed or suspected cases in the community or village	Yes	5	1.4
Relatives or friends being infected with COVID-19	Yes	7	1.9
Exposure to media coverage of the	<1 h/day	115	31.8
COVID-19	1–2 h/day	204	56.4
	>3 h/day	43	11.9
**Earthquake exposure^a^ [M (SD)]**	11.22 (2.77)		
Family member injured or	Injured	54	14.9
killed/missing	Killed or missing	42	11.6
House damage	Moderate	176	48.6
	Severe	158	43.6
Property loss	Moderate	263	72.7
	Severe	75	20.7
Directly witnessed the disaster	Yes	178	49.2

a *Measured in November 2008*.

Of the 362 participants, 24 (6.6%) had depression, with a mean PHQ-9 score of 3.01 (SD = 3.87). A total of 17 (4.7%) were shown to be positive for anxiety, with a mean GAD-7 score of 2.20 (SD = 3.29). In terms of perceived social support, only 14.4% (*N* = 52) had a low level, while 42.5% (*N* = 154) had a high level. The mean score of MSPSS was 63.98 (SD = 12.10).

Demographic characteristics along with the outcomes of interest were presented in Table 2, stratified by different levels of perceived social support. Compared to low perceived social support, participants who perceived a high level of social support were reported to have lower PHQ-9 and GAD-7 scores, as well as significantly lower proportion of depression and anxiety. Correlation analysis further showed MSPSS scores being negatively associated with PHQ-9 (*r* = −0.41, *p* < 0.001) and GAD-7 (*r* = −0.37, *p* < 0.001) scores. In addition, earthquake exposure was not associated with MSPSS (*r* = −0.07, *p* = 0.177), PHQ-9 (*r* =0.07, *p* = 0.186), and GAD-7 (*r* =0.06, *p* = 0.282) scores.

**Table 2:** Characteristics of participants enrolled to the study according to perceived social support status.

		**Perceived social support status**			***p***	**Cramer's *V***
		**Low *N* = 52, 14.4%**	**Moderate *N* = 156, 43.1%**	**High *N* = 154, 42.5%**		
Sex [*N* (%)]	Female	31 (13.1)	96 (40.5)	110 (46.4)	χ^2^ = 4.27	0.109
	Male	21 (16.8)	60 (48.0)	44 (35.2)		
Age [years, M (SD)]		26.36 (0.61)	26.40 (0.66)	26.43 (0.65)	*F* = 0.27	η^2^ = 0.022
Marital status (married) [*N* (%)]	Married	19 (12.8)	65 (43.9)	64 (43.2)	χ^2^ = 0.48	0.036
	Unmarried	33 (15.4)	91 (42.5)	90 (42.1)		
Family income (monthly) [*N* (%)]	<5,000 RMB	24 (13.4)	80 (44.7)	75 (41.9)	χ^2^ = 1.29	0.042
	5,000–10,000 RMB	17 (13.9)	50 (41.0)	55 (45.1)		
	>10,000 RMB	11 (18.0)	26 (42.6)	24 (39.3)		
History of mental disorders [*N* (%)]	Yes	0 (0)	1 (33.3)	2 (66.7)	χ^2^ = 0.91	0.050
	No	52 (14.5)	155 (43.2)	152 (42.3)		
Chronic physical illness [*N* (%)]	Yes	1 (33.3)	2 (66.7)	0 (0)	χ^2^ = 2.44	0.082
	No	51 (14.2)	154 (42.9)	154 (42.9)		
Smoking [*N* (%)]	Yes	15 (21.1)	35 (49.3)	21 (29.6)	χ^2^ = 7.09^*^	0.140
	No	37 (12.7)	121 (41.6)	133 (45.7)		
Alcohol intake [*N* (%)]	Yes	39 (17.1)	98 (43.0)	91 (39.9)	χ^2^ = 4.22	0.108
	No	13 (9.7)	58 (43.3)	63 (47.0)		
Confirmed or suspected cases in	Yes	0 (0)	2 (40.0)	3 (60.0)	χ^2^ = 1.10	0.055
the community or village [*N* (%)]	No	52 (14.6)	154 (43.1)	151 (42.3)		
Relatives or friends being	Yes	3 (42.9)	3 (42.9)	3 (14.3)	χ^2^ = 5.37^*^	0.122
infected with COVID-19 [*N* (%)]	No	49 (13.8)	153 (43.1)	153 (43.1)		
Exposure to media coverage of	<1 h/day	17 (14.8)	51 (44.3)	47 (40.9)	χ^2^ = 1.73	0.049
the COVID-19 [*N* (%)]	1–2 h/day	27 (13.2)	90 (44.1)	87 (42.6)		
	>3 h/day	8 (18.6)	15 (34.9)	20 (46.5)		
Earthquake exposure [M (SD)]	–	11.62 (2.73)	11.27 (2.92)	11.04 (2.62)	*F* = 0.89	η^2^ = 0.049
PHQ-9 [M (SD)]	–	5.87 (5.43)	3.45 (3.81)	1.60 (2.41)	*F* = 29.32^***^	η^2^ = 0.178
Depression^a^ [*N* (%)]	Yes	11 (45.8)	11 (45.8)	2 (8.3)	χ^2^ = 24.84^***^	0.262
	No	41 (12.1)	145 (42.9)	152 (45.0)		
GAD-7 [M (SD)]	–	4.17 (4.63)	2.61 (3.39)	1.10 (2.01)	*F* = 21.30^***^	η^2^ = 0.126
Anxiety^b^ [*N* (%)]	Yes	8 (47.1)	9 (52.9)	0 (0)	χ^2^ = 21.26^***^	0.242
	No	44 (12.8)	147 (42.6)	154 (44.6)		

*MSPSS, Multidimensional Scale of Perceived Social Support; PHQ-9, Patient Health Questionnaire; GAD-7, Generalized Anxiety Disorder Scale*.

a *Depression calculated using the PHQ-9, with a clinical cutoff score of 10*.

b *Anxiety calculated using the GAD-7, with a clinical cutoff score of 10*.

* *P < 0.05*;

^**^P < 0.01;

*** *P < 0.001*.

**Figure 1:**
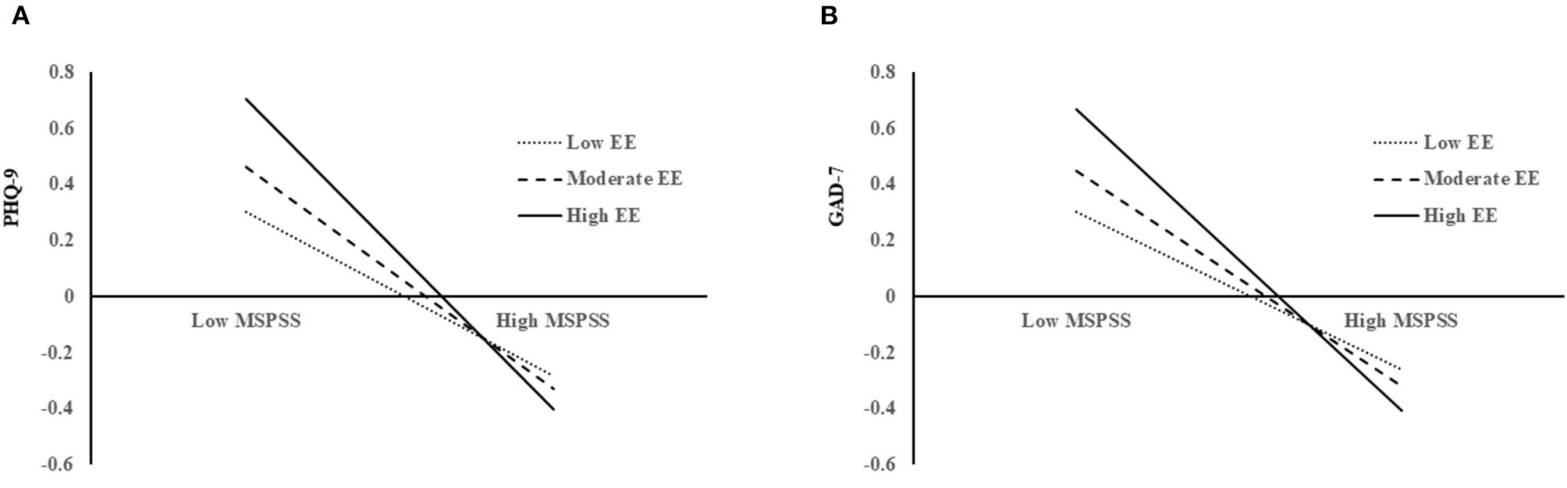
Simple slope analyses. **(A)** The interaction between perceived social support and depression as moderated by earthquake exposure and shows that perceived social support was negatively associated with depression better with higher earthquake exposure. **(B)** The interaction between perceived social support and anxiety as moderated by earthquake exposure and shows that perceived social support was negatively associated with anxiety better with higher earthquake exposure. MSPSS, Multidimensional Scale of Perceived Social Support; EE, earthquake exposure; PHQ-9, Patient Health Questionnaire; GAD-7, Generalized Anxiety Disorder Scale.

As shown in Table 3, the moderation model with PHQ-9 score as outcome was significant with *F*_(14, 347)_ = 7.40, *p* < 0.001, accounting for 23.0% of the total variance. Perceived social support had a negative main effect on depressive symptoms (*b* = −0.40, SE = 0.05, 95% CI = −0.49, −0.30). While earthquake exposure did not directly affect depressive symptoms (*b* = 0.05, SE = 0.05, 95% CI = −0.05,0.14), it moderated the relationship between perceived social support and depressive symptoms, *b* = −0.14, SE = 0.05, 95% CI = −0.24, −0.04, indicating that the indirect effect of perceived social support on depressive symptoms significantly differed at various levels of earthquake exposure. With simple slope analyses, a significant negative relationship between perceived social support and depressive symptoms was found at low (*b* = −0.28, SE = 0.06, 95% CI = −0.41, 0.16), moderate (*b* = −0.38, SE = 0.05, 95% CI = −0.48, −0.29), and high levels of earthquake exposure (*b* = −0.54, SE = 0.07, 95% CI = −0.67, −0.40) (see Figure 1A).

**Table 3:** Regression coefficients from analyses of moderating effect of earthquake exposure on the relationship between perceived social support and mental health status.

	***b***	**SE**	***t***	***p***	**95% CI**
**Model 1^a^**
MSPSS	−0.40	0.05	−8.10	<0.001	−0.49, −0.30
EE	0.05	0.05	1.01	0.313	−0.05, 0.14
MSPSS × EE	−0.14	0.05	−2.86	0.005	−0.24, −0.04
Low EE	−0.28	0.06	−4.40	<0.001	−0.41, −0.16
Moderate EE	−0.38	0.05	−7.81	<0.001	−0.48, −0.29
High EE	−0.54	0.07	−7.93	<0.001	−0.67, −0.40
**Model 2^b^**
MSPSS	−0.38	0.05	−7.70	<0.001	−0.48, −0.28
EE	0.04	0.05	0.66	0.511	−0.06, 0.13
MSPSS × EE	−0.14	0.05	−2.76	0.006	−0.24, −0.04
Low EE	−0.27	0.07	−4.16	<0.001	−0.40, −0.14
Moderate EE	−0.37	0.05	−7.42	<0.001	−0.47, −0.27
High EE	−0.52	0.07	−7.57	<0.001	−0.66, −0.39

*Models both adjusted for sample characteristics (e.g., sex and age) and pandemic-related factors*.

*MSPSS, Multidimensional Scale of Perceived Social Support; EE, earthquake exposure; OR, odds ratio; CI, confidence intervals*.

a *PHQ-9 score was the outcome*.

b *GAD-7 score was the outcome*.

The moderation model with GAD-7 score as the outcome was also significant with *F*_(14, 347)_ = 6.40, *p* < 0.001, accounting for 20.5% of the total variance. Similar to the results of depressive symptoms, earthquake exposure moderates the relationship between perceived social support and anxiety symptoms (*b* = −0.14, SE = 0.05, 95% CI = −0.24, −0.14). Simple slope analyses also found a significantly positive relationship between perceived social support and anxiety symptoms at low (*b* = −0.27, SE = 0.07, 95% CI = −0.40, −0.14), moderate (*b* = −0.37, SE = 0.05, 95% CI = −0.47, −0.27), and high (*b* = −0.37, SE = 0.07, 95% CI = −0.66, −0.39) level of earthquake exposure (see Figure 1B). These findings indicated that earthquake exposure is a moderator of perceived social support and mental health among earthquake survivors during the COVID-19 outbreak.

## Discussion

To our best knowledge, this was the first study to examine the mental health status of prior natural disaster survivors during the COVID-19 outbreak. Our findings suggested that earthquake survivors did not confer any increase in the prevalence of depression and anxiety during the outbreak phase of the pandemic. Meanwhile, higher prior earthquake exposure experience strengthens the protective effect of individual's perceived social support.

This study found that only 6.6% of the participants reported having depression and 4.7% reported having anxiety. In order to understand the meaning of our results, they are compared with data of the same type (the PHQ-9/GAD-7 cutoff of 10 or higher) on national and international surveys during the pandemic outbreak. Based on previous research, depression and anxiety rate in the current sample was lower than that of a similar study measuring Chinese adult citizens (12% depression, 7.1% anxiety) between February 9 and 20, 2020 (36). Several web-based studies found that the percentage of Chinese general public with depression was 13.6% between February 11 and 16, 2020 (37), and anxiety rate was 22.6% between January 31 and February 2, 2020 (38). Meanwhile, the general population from Jordan reported 32.1% depression and 22.8% anxiety between March 22 and 28, 2020 (39). Ettman et al. observed that the prevalence rate of depression was 27.8% in U.S. general adults during the COVID-19 outbreak (March 31–April 13, 2020) (40). Compared to these studies that have taken place in a similar phase of the outbreak, lower levels of depression and anxiety symptoms were observed in earthquake survivors.

Our study also found a higher level of perceived social support in earthquake survivors when compared to that of general college students in China during the same period (February 3–10, 2020). Our results showed that perceived social support of the present sample (mean score = 63.98, SD = 12.10) was higher than the level of college students (mean score = 59.8, SD = 11.7) living in the moderate-risk (Guangdong Province) and low-risk aeras (Jiangxi Province) (41). In the current sample, 42.5% of participants could be classified into the high social support group (scores from 69 to 84), which seemed to be significantly higher than the rate in the Lebanese public (20.8%) during the outbreak of COVID-19 with a consistent demarcation (42). Earthquake survivors having higher perceived social support in our study may be due to the solid financial and emotional support from both the government and the civilians in China (43), such as house reconstruction and better healthcare. In addition, perceived social support was observed to have a significant negative association with anxiety and depression. Higher levels of perceived social support were related to lower level of depression and anxiety outcomes, which was in line with previous literature (42, 44). It has been proposed that such social support could predict better mental health functioning and be regarded as a protective factor against the onset of new mental health problems (12, 45). More specifically, social support could also enhance resilience to stress and reduce the development of trauma-related psychopathology (14).

Interestingly, prior earthquake exposure did not exhibit a direct effect on current depressive and anxiety symptoms, but it moderated the relationship between perceived social support and psychological symptoms. The effect of perceived social support on depression or anxiety significantly differed at varying levels of prior earthquake exposure. Specifically, social support had a stronger protective effect on mental health among survivors who had greater earthquake exposure. Although scholars proposed that trauma was a vital risk factor for individuals' mental health issues (46), prior trauma exposure might also have salutogenic effects. Recent evidence found that people with high trauma exposure were more likely to experience posttraumatic growth (PTG) (47), which denoted the tendency to report a positive transformation in the aftermath of a trauma exposure (48). Scrutinizing the empirical literature also found that participants with higher PTSD symptoms were more likely to grow from the impact of the trauma (49, 50). Theoretically, PTG might indicate perceived change rather than reflect actual growth (51). It could also be understood as a motivated positive illusion that served a protective function (52). We speculated that these improved personal resources and qualities [e.g., resilience (53)] that precipitated from past adversities acted as active protective factors that could be set in motion as one facing adversities again (e.g., COVID-19 pandemic).

Finally, several limitations must be considered. First, the present study was conducted on a sample of trauma survivors who experienced the 2008 Wenchuan earthquake. Before the disaster, they were both students of junior and senior school (Grades 7–12). Therefore, generalizations of our findings to sufferers of other traumatic experiences or of different age groups need to be done with caution. Second, there was a high attrition rate in the present study, which may lead to affect the accuracy of results. The time interval between the two surveys was more than 11 years, resulting in a high attrition rate. Although no significant differences were found for earthquake exposure between participants who followed up and those lost to follow-up, the results need to be interpreted with caution. Third, depression and anxiety variables relied on self-report questionnaires, which might cause potential reporting bias in the data collection. Meanwhile, other important factors that might affect the study findings, such as PTG or actual support, were not examined. In addition, depression and anxiety among the current sample needed to be further assessed longitudinally. Mental healthcare should still be provided to those prior trauma survivors at risk in the aftermath of the pandemic.

## Conclusion

In conclusion, this study described the unique contribution of prior trauma exposure in explaining trauma-related symptoms among earthquake survivors during the COVID-19 outbreak. Earthquake survivors seemed to perceive higher levels of social support and exhibit lower mental health problems. They might also have a faster decline in mental health problems if they have been involved in greater prior trauma.

## Data Availability Statement

The raw data supporting the conclusions of this article will be made available by the authors, without undue reservation.

## Ethics Statement

The studies involving human participants were reviewed and approved by the Ethics Board of the South China Normal University. The patients/participants provided their written informed consent to participate in this study.

## Author Contributions

DW, FF, and XL: conceptualization. DW: methodology, formal analysis, and writing—original draft. DW, JC, YC, SH, and CW: data curation. SZ, FF, and XL: writing—review and editing. All authors contributed to the article and approved the submitted version.

## Funding

The present study was funded by the National Natural Science Foundation of China (Grant No. 31871129); Research on the Processes and Repair of Psychological Trauma in Youth, Project of Key Institute of Humanities and Social Sciences, MOE (Grant No. 16JJD190001); Guangdong Province Universities and Colleges Pearl River Scholar Funded Scheme (GDUPS 2016); and Graduate Research and Innovation Project of School of Psychology, South China Normal University (PSY-SCNU202017).

## Conflict of Interest

The authors declare that the research was conducted in the absence of any commercial or financial relationships that could be construed as a potential conflict of interest.

## Publisher's Note

All claims expressed in this article are solely those of the authors and do not necessarily represent those of their affiliated organizations, or those of the publisher, the editors and the reviewers. Any product that may be evaluated in this article, or claim that may be made by its manufacturer, is not guaranteed or endorsed by the publisher.
